# Probabilistic Assessment of Above Zone Pressure Predictions at a Geologic Carbon Storage Site

**DOI:** 10.1038/srep39536

**Published:** 2016-12-20

**Authors:** Argha Namhata, Sergey Oladyshkin, Robert M. Dilmore, Liwei Zhang, David V. Nakles

**Affiliations:** 1 Department of Civil & Environmental Engineering, Carnegie Mellon University, Pittsburgh, PA 15217, USA; 2 U.S. Department of Energy, National Energy Technology Laboratory, 626 Cochrans Mill Road, Pittsburgh, Pennsylvania 15236, USA; 3 Department of Stochastic Simulation and Safety Research for Hydrosystems (IWS/SRC SimTech), University of Stuttgart, Germany

## Abstract

Carbon dioxide (CO_2_) storage into geological formations is regarded as an important mitigation strategy for anthropogenic CO_2_ emissions to the atmosphere. This study first simulates the leakage of CO_2_ and brine from a storage reservoir through the caprock. Then, we estimate the resulting pressure changes at the zone overlying the caprock also known as Above Zone Monitoring Interval (AZMI). A data-driven approach of arbitrary Polynomial Chaos (aPC) Expansion is then used to quantify the uncertainty in the above zone pressure prediction based on the uncertainties in different geologic parameters. Finally, a global sensitivity analysis is performed with Sobol indices based on the aPC technique to determine the relative importance of different parameters on pressure prediction. The results indicate that there can be uncertainty in pressure prediction locally around the leakage zones. The degree of such uncertainty in prediction depends on the quality of site specific information available for analysis. The scientific results from this study provide substantial insight that there is a need for site-specific data for efficient predictions of risks associated with storage activities. The presented approach can provide a basis of optimized pressure based monitoring network design at carbon storage sites.

Capture and geologic storage of carbon dioxide (CO_2_) is considered as one of a portfolio of solutions for the reduction of anthropogenic greenhouse gas emissions. The increasing emphasis on the commercialization and implementation of CO_2_ capture and storage (CCS) has led to the development of system-wide mathematical models for the quantitative assessment of system performance and the risk associated with it. A major technical and regulatory concern that has gained attention of the research community is the unanticipated leakage of CO_2_ and brine from deep storage reservoirs to overlying geologic formations such as the Above Zone Monitoring Interval (AZMI) and groundwater aquifers^1^,^2^,^3^, through preferential migration pathways such as, wellbores, faults, fractures and presence of high-permeability zones in the caprocks. While the leaked CO_2_ and brine may possess threat to the environmental receptors, it is also possible that it would attenuate pressure and CO_2_ saturation. The knowledge of changes in pressure helps for adequate management of the reservoir. Hence, it becomes important to monitor the above zone pressure, since it may provide potentially useful source of information about seal performance and subsurface pressure response to CO_2_ and brine leakage from the storage reservoir.

The United States Department of Energy (DOE) through its National Risk Assessment Partnership (NRAP)^4^,^5^, is conducting research to develop and demonstrate science-based methodologies to quantify the environmental risks associated with long-term geologic storage of CO_2_^6^. Central to this research is the development of Integrated Assessment Models, or IAMs to describe site-scale performance of geologic storage systems. These IAMs are system-based models that simulate and couple the primary sub-system components of the storage system, i.e., storage reservoir, migration pathways (i.e., seals, wellbores, faults and fractures), groundwater, and atmosphere, with the goal of predicting potential leakage performance/storage security through the period of active CO_2_ injection, and post-injection site care. Since the integration of fully characterized numerical models of individual sub-system into an IAM is both challenging and computationally expensive, the NRAP approach calls for modeling the sub-system components using simplified reduced order characterizations, or reduced order models (ROMs), that are much more computationally efficient^3^,^5^,^7^,^8^,^9^,^10^,^11^,^12^,^13^.

One such ROM development effort has focused on characterizing the CO_2_ and brine leakage through the primary sealing layer to the interval directly overlying that seal (the AZMI). A ROM developed and previously reported by Namhata and coworkers^14^ predicts spatially-varying changes in pressure through time in response to that fluid leakage. The AZMI ROM is believed to provide a useful approximation of real-world response, but also includes a number of conceptual and quantitative uncertainties. Insufficient or lack of information related to geological properties represents one important source of parameter uncertainty that may lead to significant uncertainties in model predictions, with potential to mask the influence of secondary physical processes^15^. Because full-physics numerical simulation models are computationally expensive, it may require several hours to days to complete a single, deterministic realization; as such, exploring uncertainty/variability in system performance using such models and brute-force Monte Carlo simulation is generally considered intractable^16^,^17^,^18^,^19^,^20^. This makes it favorable to use advanced stochastic tools to model uncertainties of complicated processes involved in the geologic storage of carbon modeling. It also holds true for a coupled ROM approach. Application of advanced stochastic tools to predict uncertainties in coupled ROM systems like the reservoir-caprock-AZMI coupled model used in this study will be computationally efficient over a complex Monte Carlo like analysis.

In this study, we use a recently developed data-driven uncertainty quantification approach, called the arbitrary polynomial chaos (aPC) expansion that provides a massive stochastic model reduction^15^,^21^ to analyze the uncertainties in predictive ability of the AZMI ROM. aPC has certain advantages over more conventional polynomial chaos methods. This approach provides a more robust convergence^21^ in comparison to the classical methods (e.g., Wiener, 1938; Ghanem and Spanos, 1993; Le Maître and Knio, 2010)^22^,^23^,^24^ once underlying distributions of uncertain parameters dictated by real-world data; it also allows for use of arbitrary probability distributions of uncertain parameters^21^. The more complex the system is, the greater will be the associated uncertainty of the system models. Uncertainty of any parameter in the modeling procedure propagates through the model to impact the model predictions. Hence, it is important to rank the influence of the model input parameters on the output space. This aids in better understanding the system behavior, adding value to the task of analyzing model uncertainties and sensitivities.

Sensitivity analysis is widely used to identify the contribution of uncertainty sources within the modeling process^21^ and that in turn helps in improving the understanding of model behavior^25^. We performed global sensitivity analysis (GSA) using variance-based Sobol sensitivity index parameterization^25^. The motivation to GSA over a local sensitivity analysis approach is that local analysis is unable to cover the non-linear variation of model responses over the entire range of probability distributions of the input parameters^26^. The aim of GSA is to quantify the relative importance of each individual input parameter on model output prediction, and rank those parameters by importance. The aPC-based response surface used in the uncertainty quantification is based on orthonormal polynomials whose properties are well exploited^27^. The goal of this study is to probabilistically assess the role of various geologic parameters in AZMI pressure predictions.

## Results and Discussions

### Above Zone Model Setup

**Figure 1:**
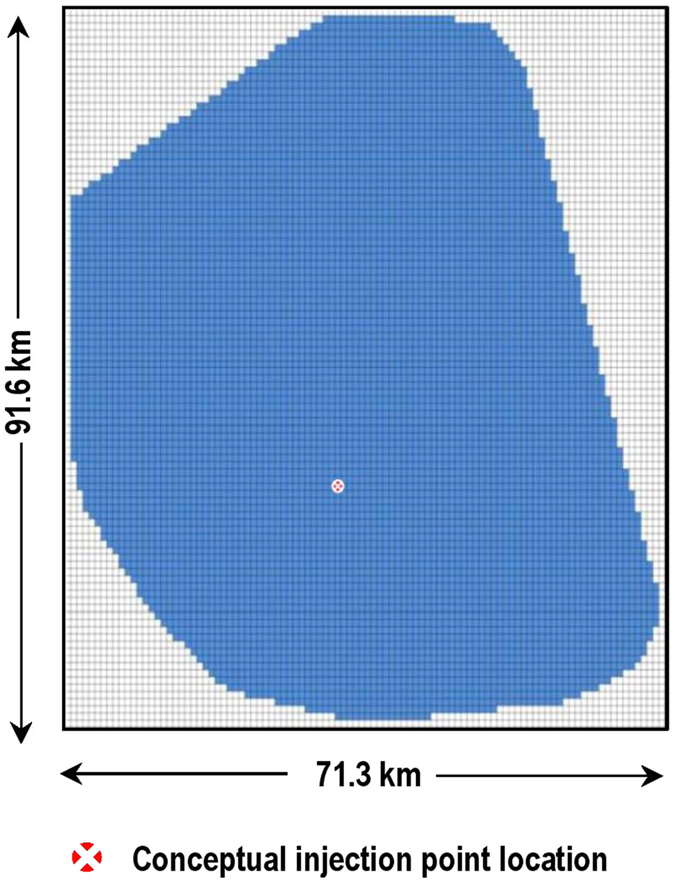
Plan view of the model domain (in blue) with numerical grid. The red point is the location of the conceptual injection well (coordinate (34, 46)).

In this modeling effort, we aim to study the migration of subsurface fluids (here, CO_2_ and brine) to the AZMI and the resulting changes in pressure. The modeled system comprises three components: reservoir, caprock and the AZMI. The calculations in reservoir and caprock are necessary to model the pressure changes in the AZMI. This study demonstrates the application of above zone pressure modeling using the AZMI ROM by using the Kimberlina CO_2_ storage site (California, USA) as an example^28^,^29^,^30^,^31^. The reservoir-scale CO_2_ migration model developed by Wainwright *et al*.^30^ is based on a geological study in the Southern San Joaquin Basin, California. The model uses geologic and hydrogeologic data obtained from many oil fields in that region. The model domain extends 71.3 km in the eastern direction and 91.6 km in the northern direction as shown in Fig. 1. The simulation assumes that CO_2_ injection is conducted in the center of the domain into the 400-m thick and at about 2750 m deep Vedder formation (depth is at top). The Vedder formation is quite permeable which should allow large industrial scale fluid injectivity. The injection well location is also marked in the Fig. 1. Since we intend to use the reservoir simulator results in seal ROM, NSealR^7^, the reservoir area is converted into 100 by 100 grid block system, for consistency. The location of the conceptual injection well is at coordinate (34, 46) in that reduced-resolution spatial domain. The overlying Temblor–Freeman shale with a nominal thickness of 200 m is considered a suitable caprock for stratigraphic containment of the supercritical CO_2_ injected into the underlying Vedder formation. This storage formation site model is used because there are considerable data available. In this paper, we use the modified Kimberlina model of Wainwright *et al*.^30^ as used by Pawar and co-authors^4^ to simulate the reservoir pressure and saturations. A hypothetical scenario is assumed where 5 million tons of CO_2_ is injected per year for a period of 50 years. There are several faults known to be present in the reservoir. Fault zone properties are quite uncertain; however, there are qualitative observations that most fault zones are less conductive than the adjacent sandstone formations^17^. In the reservoir simulations, the potential for leakage of CO_2_ and/or brine through permeable faults and/ or fractures has been ignored, i.e., the seal is characterized with realizations of uniform, but varying permeability. Also, the potential for fault reactivation in response to fluid injection is not addressed. Since there is no information about the fault zones, we just assumed that there are no faults transcending into the caprock and/or above layers. Supplementary Figs S1 and S2 show the pressure and saturation at the reservoir-seal interface, respectively. It can be seen from Fig. S1 that the pressure increase spreads from the injection zone to the boundaries of the domain during the 50 year CO_2_ injection period, and the pressure increase gradually decreases once the injection stops. Figure S2 shows that when compared to pressure, the CO_2_ plume is more localized, and the observable increase in CO_2_ saturation can only be seen right above the injection zone. After the reservoir simulation, we use the NRAP Seal Barrier ROM, NSealR^7^ to compute the migration of CO_2_ and brine through the seal to overlying AZMI formation through intrinsic permeability and/or the presence of natural/induced fractures in the seal. NSealR uses a two-phase, relative permeability approach with Darcy’s law for one-dimension (1-D) flow computations of CO_2_ through the horizon in the vertical direction. The reservoir pressure and saturation generated using the Kimberlina model^30^ is used as an input to NSealR to produce CO_2_ and brine flux from top of the seal in a 100 by 100 uniform grid format. The CO_2_ flux through the 200-m thick Temblor–Freeman shale calculated using NSealR is shown in Supplementary Fig. S3. Although the case considered assumes a single thickness seal, NSealR allows for spatially varying thickness and effective permeability.

The AZMI ROM used in this study to predict above zone pressure changes due to leakage through the primary seal has been previously described by Namhata *et al*.^14^. A hypothetical AZMI system for Kimberlina is defined for the model analysis. This conceptual base case system consists of a 10-m thick AZMI layer overlying the aforementioned Temblor–Freeman shale of thickness 200 m. The AZMI formation features have been derived from the existing Olcese sandstone which overlies the Temblor-Freeman shale. It is assumed that the AZMI is initially fully saturated with brine. The reference parameters used for the Kimberlina site in this model are taken from Wainwright *et al*.^30^ and are shown in Table 1. Relative permeability has important implications for fluid flow in subsurface geological systems. The Brooks-Corey^32^ model has been used to define relative permeability in the AZMI ROM. The relative permeability curve used for this study has been shown in the Supplementary Fig. S4.

**Figure 2:**
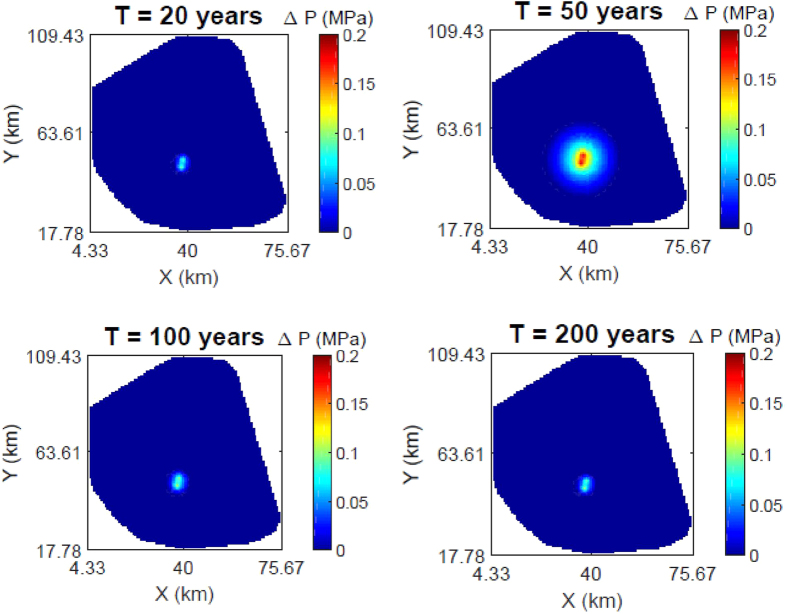
Changes in pressure response (in MPa) at the top of the AZMI at 20, 50, 100 and 200 years after the start of CO_2_ injection (base case results).

Figure 2 presents the changes in pressure response in the AZMI over time generated using flux from the seal for the simulation periods previously discussed. The highest increase in pressure in the AZMI is observed above the injection point at the end of injection (i.e., 50 years), with the maximum predicted increase in pressure of 0.185 MPa. The change in pressure gradually decreases away from the injection point location. After CO_2_ injection stops, the rate of increase in pressure abruptly stops with the termination of injection, followed by an initial fast rate of pressure decrease that gradually slows and approaches a stable, but positive net pressure change by the end of the simulation (i.e., following a post-injection period of 150 years). Supplementary Fig. S5 shows the time evolution of pressure change for the base case.

### Uncertainty Quantification of Above Zone Pressure

Many geologic parameters are influential in predicting the CO_2_ and brine flow dynamics in the AZMI ROM. Due to lack of information stemming from a limited ability to make direct measurements, parameters such as porosity and permeability, to name two, often remain uncertain^33^,^34^. These uncertainties can have a substantial effect on the output of the ROM. Thus, a quantitative analysis of the impact of these uncertainties on the predictive capabilities of the model was performed and is presented in this section.

A model-based uncertainty analysis, though efficient, requires statistical data for all of the model input parameters, which increases the demand on data availability or results in highly subjective assumptions to deal with missing data^15^. Uncertainties in complex systems can be efficiently and accurately quantified using stochastic models based on an approach using data-driven polynomial chaos expansion (PCE) methods^35^,^36^,^37^,^38^. Thus, the uncertainty quantification of the AZMI ROM was performed using the arbitrary Polynomial Chaos (aPC) approach^15^,^21^. In aPC, the statistical moments are the only source of information required to define the stochastic parameters. Hence, accurate descriptions of the probability density functions (PDF) of the uncertain parameters are not required to perform the analysis.

### Statistical Distribution of Input Parameters

The data-driven aPC method, as described in the Methods section, only requires information on finite number of moments, and does not explicitly require the shapes of probability density functions. The arbitrary distributions can be either discrete, continuous, or discretized continuous and can be specified either through a few statistical moments, analytically as PDF/CDF, numerically as a histogram, or theoretically through the even more general format of a probability measure^15^.

**Figure 3:**
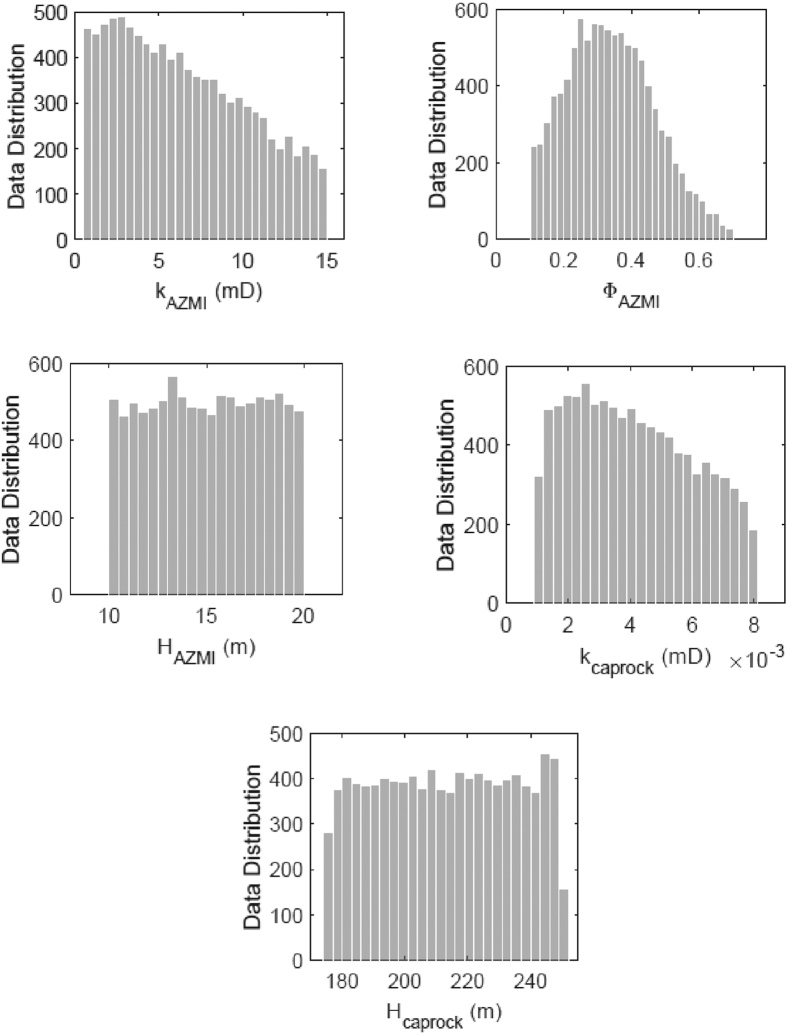
Distribution of AZMI permeability (k_AZMI_), AZMI porosity (Φ_AZMI_), thickness of AZMI (H_AZMI_), caprock permeability (k_caprock_) and caprock thickness (H_caprock_) for aPC uncertainty analysis.

In this study, the uncertainty analysis was performed for five input parameters: AZMI permeability (k_AZMI_), AZMI porosity (Φ_AZMI_), thickness of AZMI (H_AZMI_), caprock permeability (k_caprock_) and caprock thickness (H_caprock_). Though the study is site specific, caprock and AZMI thickness are considered to be a part of the list of uncertain parameters. The purpose of using these parameters is to have an idea of how the uncertainty will be in a generalized setup in case there is not enough information about the formation thickness. Also, the thickness of formation usually doesn’t tend to be uniform throughout storage sites and hence this assessment will be helpful in finding out the effect of thickness on pressure buildup. Figure 3 demonstrates the stochastically generated distributions of the parameters that have been used in the analysis. The data distribution pattern is generated based on data available from the US National Petroleum Council Public Database^38^,^39^.

### AZMI Output Statistics

**Figure 4:**
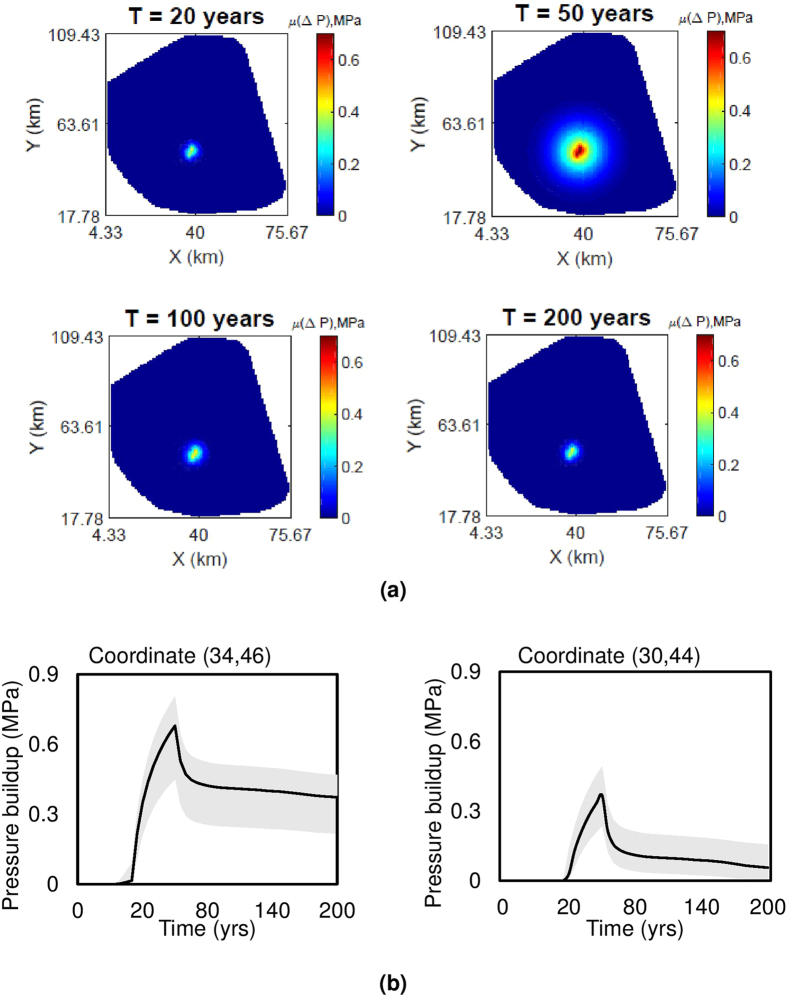
Plots showing (**a**) mean change in pressure response (in MPa) at the top of AZMI over time based on aPC analysis, (**b**) mean pressure change (black line) and range of pressure change from 56 simulations (grey shaded region) above injection point, i.e., coordinate (34, 46) and 4 km away from injection well southwards, i.e., coordinate (30, 44) over time.

**Figure 5:**
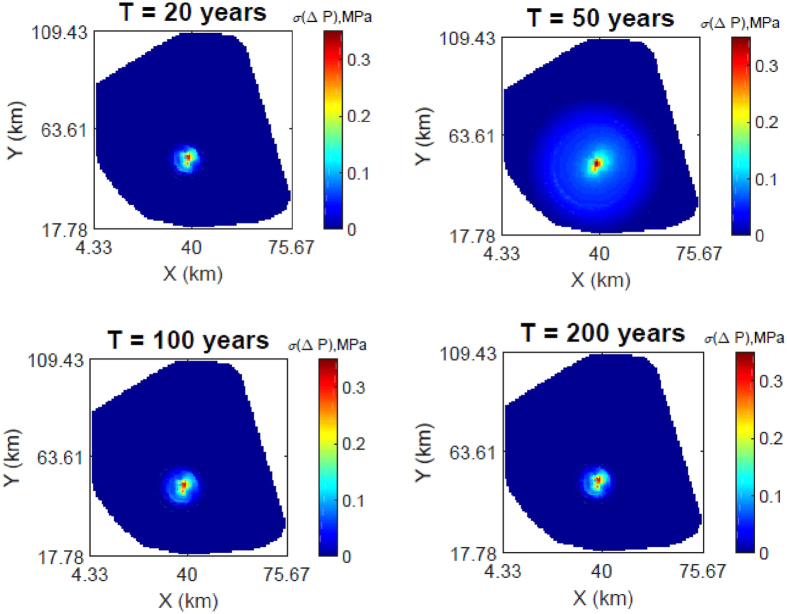
Estimation of standard deviation of the change in pressure response (in MPa) prediction by AZMI ROM at the top of AZMI.

**Figure 6:**
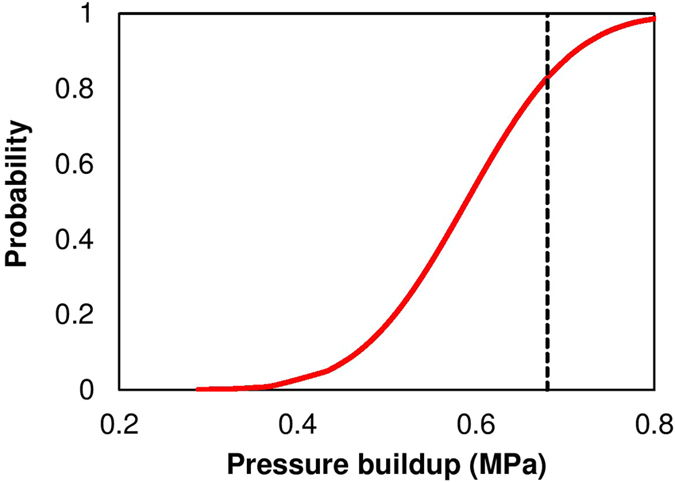
Probability of detection of pressure build-up (shown in red line) at the top of the AZMI above injection point, i.e., coordinate (34, 46) at the end of injection. The black dotted line shows the mean of pressure build-up based on aPC analysis.

We analyze these statistical moments for the AZMI ROM for the simulation period of 200 years. Mean and standard deviation of changes in pressure response above the AZMI over time is shown in Figures 4(a) and 5 respectively, based on the uncertainty of the five input parameters. A total of 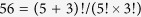 detailed simulations (see equation (3)) is carried out to generate the uncertainties in model outputs based on aPC framework. It can be seen from Fig. 4(a) that the mean of pressure buildup above the AZMI from the aPC simulations is approximately 0.50 MPa higher than that of the base case scenario in Fig. 2. Since the highest-pressure buildup above the AZMI occurs right above the injection well location, we checked the variation in pressure change output from the entire set of simulations to that of the calculated mean. The analysis is shown in Fig. 4(b) by plotting the range of predictions from the 56 simulations. We also estimate the probability of detecting a pressure build-up above the injection well using cumulative distribution plot. Figure 6 shows the probability distribution of pressure build-up above the injection point. This result can be used to predict the risk associated with CO_2_ leakage at the AZMI. If the system is required to be assessed based on a threshold AZMI pressure, the probability of failure can be calculated based on such results. It can be seen from Fig. 5 that the areal extent of standard deviation increases as more CO_2_ is injected into the system. Then the deviation starts to decrease considerably over time. The maximum deviation in pressure change is also above the injection well location. It is consistent with the fact that the greater the amount of leakage, the greater will be the pressure buildup. Thus it becomes important to gather high-accuracy data of the geologic properties. Uncertainties of input parameters can lead to significant deviations in model outputs. Hence, the need for site-specific data is an essential requirement for efficient model predictions which validates what geoscientists know. Larger variation in input parameters of a model will lead to large deviations in outputs, which will lead to failure in understanding of the storage system and hence predict the containment risk properly. This work shows a parameter based uncertainty. Model based uncertainty analysis (e.g., Goodman *et al*.^40^) can also be carried out by comparing predictions for similar simulation settings using other coupled reservoir-caprock-AZMI models to account for the uncertainty in model representativeness. The large uncertainties in the AZMI ROM prediction makes it important to analyze the role of each individual parameter on the output space. Therefore, we do a sensitivity analysis of the AZMI ROM, shown in the following section.

### Sensitivity Analysis of Modeling Parameters

Assessment of the relative importance of the input parameters on the AZMI ROM output is required to understand the degree of their individual impact on the model predictions. This assessment is performed using a global sensitivity analysis with Sobol indices that are based on the aPC technique as described in Ashraf *et al*.^26^ and Oladyshkin *et al*.^27^, as described in the Methods section. As discussed in these previous works, the global aPC-based sensitivity analysis obtains global sensitivity information at low computational costs.

**Figure 7:**
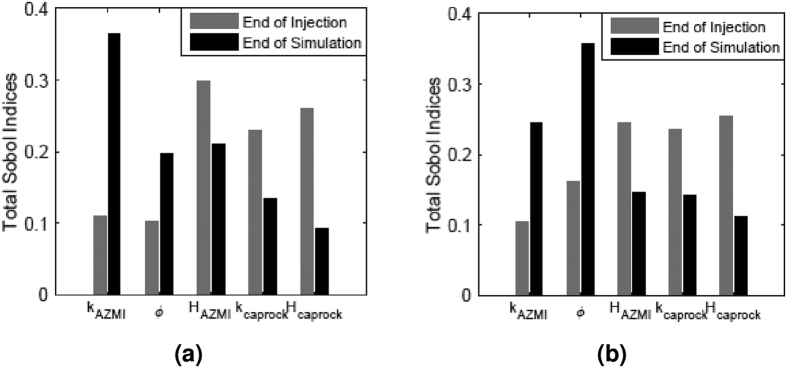
Sensitivity of AZMI ROM output for changes in pressure with respect to the uncertain parameters: AZMI permeability (k_AZMI_), AZMI porosity (Φ_AZMI_), thickness of AZMI (H_AZMI_), caprock permeability (k_caprock_) and caprock thickness (H_caprock_) at: (**a**) above the injection well, i.e. coordinate (34, 46) and, (**b**) 4 km away from injection well southwards, i.e., coordinate (30, 44).

Quantitative sensitivity information for the AZMI ROM is extracted from the polynomial response surface. The Sobol indices (equation (10)) and the total Sobol indices (equation (11)) calculations are being done for the AZMI modeling scenario. The results are based on the 3^rd^ order aPC expansion being assessed by fifty-six detailed simulations carried out for the uncertainty analysis. Model sensitivity analysis is performed for the five previously described input parameters (i.e., AZMI permeability, AZMI porosity, AZMI thickness, caprock permeability and caprock thickness) that have been used to quantify the model uncertainty. The test evaluated the impact of these parameters on the model output–pressure buildup response. The total Sobol sensitivities of input parameters on the ROM output are summarized in Fig. 7. The figure presents the sensitivity results above the injection point and a point approximately 4 km down south from the injection point at the end of injection period (=50 years) and at the end of simulation (=200 years). Table 2 represents the ranked 2^nd^ order Sobol indices for the five uncertain parameters at the end of injection (=50 years). The Sobol sensitivity calculation is shown for the 2^nd^ order expansion and not the 3^rd^. The results are shown just to represent how parameter-parameter interaction can play a role in sensitivity calculation.

**Figure 8:**
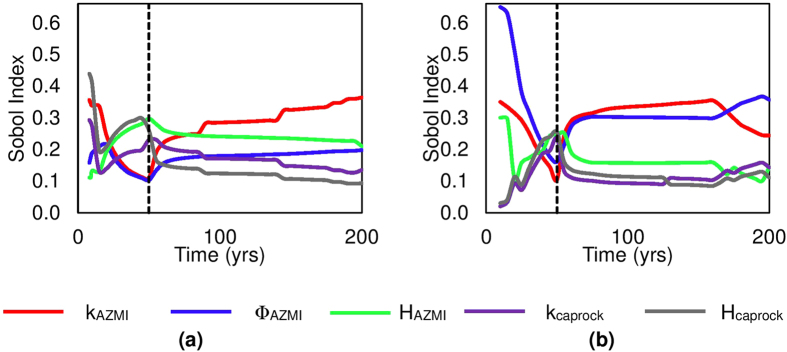
Sobol sensitivity results for AZMI ROM outputs over time at (**a**) above the injection well, i.e. coordinate (34, 46) and, (**b**) 4 km away from injection well southwards, i.e., coordinate (30, 44).

Figure 8 shows the time profile of the total Sobol indices (*S*_*Ti*_) measured using equation (11), quantifying the contribution of a modeling parameter on the uncertainty of the pressure: (a) above the injection well, i.e. coordinate (34, 46) and, (b) 4 km away from injection well southwards, i.e., coordinate (30, 44). The sensitivity is normalized by variance at each time step. It should be noted that the sum of *S*_*Ti*_ of each parameter need not be equal to one, suggesting the presence of parameter-parameter interaction effects^41^. The AZMI permeability (k_AZMI_) is clearly the most influential parameter, with higher total Sobol index corresponding to higher pressure buildup. If the permeability of the AZMI is high, pressure should easily dissipate resulting in lower pressure buildup in the AZMI. If the formation has a higher porosity, it means it can store more CO_2_ per unit volume of the porous medium. This allows the incoming CO_2_ to later accumulate the porous space and causing pressure change in the area. When the flow physics changes from injection to a gravity-dominated system, we observe a distinct change in the sensitivity patterns. During the injection period, H_AZMI_, k_caprock_ and H_caprock_ are more dominant than k_AZMI_ and Φ_AZMI_. The reason is that the incoming CO_2_ takes time to mobilize and accumulate in the AZMI. Initially the model is largely dominated by the incoming flux through the seal which is dependent on k_caprock_ and H_caprock_ and the pressure buildup is also positively affected by the thickness of the AZMI, H_AZMI_. Higher permeability of caprock leads to higher CO_2_ and brine mobility, which leads to higher pressure buildup in the AZMI from incoming CO_2_ and brine. The sensitivity of the pressure output with respect to higher AZMI permeability jumps up, right after stopping the injection.

## Methods

### Arbitrary Polynomial Chaos Expansion

Assuming a physical model,


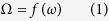


where, 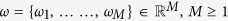 is a vector of uncertain parameters (model inputs), and, 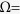

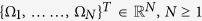
 is a vector of model outputs of interest.

The model output is a random variable if the parameter vector *ω* is uncertain. In our study the model output is a function of saturation and pressure. Polynomial chaos theory has a long history and according to Wiener, 1938^22^, Ω can be expressed in the following form:


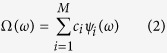


where, *c*_*i*_’s are coefficients quantifying the dependence of model output on its input and *ψ*_*i*_(*ω*) are orthogonal polynomial forming basis in the input probability space.

Since the AZMI ROM is space-time dependent, the model output is written as Ω(*X, ω*) where the vector *X* = {*x, y, t*} consists of two space coordinates and time. Hence, coefficients, *c*_*i*_ is determined for each point in space and time, i.e., *c*_*i*_(*X*).

In practice, this PCE is truncated at a finite number of basis functions, *ψ*_*i*_. The number of the terms *M* in equation (2) depends on the total number of input parameters *N* and the order *d* of the expansion, i.e., the highest degree of polynomial basis functions, according to the following:


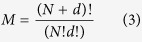


In the current study, we choose 3^rd^ order aPC expansion. We use a 3^rd^ order of expansion since it has a freedom to describe non-monotonic behaviors in comparison to the 2^nd^ order. Also, the choice of 3^rd^ problem is supported by the work of Oladyshkin and co-workers^42^ where the authors have shown convergence analysis of aPC-based Sobol analysis concluding that all expansion beyond 2^nd^ can capture non-linearity of a model. The detailed model description is provided in the Supplementary section.

### Uncertainty Quantification

Uncertainty analysis using PCE can be typically characterized using two methods: intrusive and non-intrusive. In the present context, non-intrusive probabilistic collocation method (PCM)^36^,^43^ is used, since it evaluates the coefficients in model expansion using a small number of model simulations and requires no manipulation using partial differential equation^7^. The method requires computing model Ω with *M* different sets of parameters *ω* that are called collocation points. In the current study, we use the recent version of PCM as described in Oladyshkin *et al*. 2011a^17^ to compute the collocation points.

The model outputs Ω(*ω*) are directly based on the model and the specified distribution of input parameters. The mean value (*μ*) and standard deviation (*σ*) of Ω(*ω*) are given by the following analytical relations:


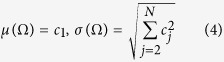


Likewise, all other moments of Ω can be obtained analytically, based on expansion coefficients and the moments of input parameters. The uncertainty outputs are space-time dependent, hence they are written as *μ*(*x, y, t*) and *σ*(*x, y, t*).

### Sobol Sensitivity Indices

A variance-based sensitivity analysis approach by calculation of Sobol Sensitivity Indices^25^ was used. Studies on the combination of PCE techniques with Sobol indices have been performed in several previous studies^27^,^44^,^45^. The basic idea behind this approach is to replace the analyzed system with an approximating function that permits the calculation of numerical and mathematical benefits of a sensitivity analysis^26^. Since the calculation of output variances from statistics of input variables of polynomials is relatively fast, polynomials are used for the approximation. For the AZMI modeling scenario, the solution is approximated by orthogonal polynomials with ascending polynomial degree.

Let us assume we break the system output into components as follows:


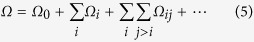


where, indices *i* and *j* show dependency on two or more variables. If we consider the input vector *ω* to have *n* component *ω*_*i*_ for *i* = *1, ……, n,* then, Ω_*i*_ = *f*_*i*_(*ω*_*i*_) and Ω_*ij*_ = *f*_*ij*_(*ω*_*i*_, *ω*_*j*_). Saltelli *et al*.^41^ defined the higher order sensitivity index, or Sobol index^46^, representing the significance of variation in output generated from the joint uncertainty in several input variables, i.e., from the interaction of uncertain parameters, as:


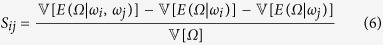


where, 
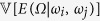
 is the variance of output expectations for a gi ven value of inputs *ω*_*i*_ and *ω*_*j*_. If all the indices containing a given variable *ω*_*i*_ are added, we get total Sobol index^15^:


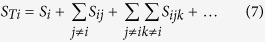


The total Sobol index is a sensitivity measure to rank parameters according to their influence on model output. The higher the index, the greater is the effect of the corresponding input parameter on the model output. Sobol indices are calculated analytically^27^ from the expansion coefficients of the aPC, shown in equation (2).

## Conclusions

This work presents the application of reduced order models (ROMs) to predict the pressure response in the Above Zone Monitoring Interval (AZMI) and flux response above the caprock using the hypothetical Kimberlina CO_2_ storage site (California, USA) as a base case example problem. We presented a data-driven arbitrary polynomial chaos expansion (aPC) method for uncertainty and sensitivity analysis of above zone pressure predictions. The data-driven approach provides a response surface based on a global orthonormal polynomial basis for arbitrary distributions. The method does not require extensive statistical knowledge for the data analysis. Thus, the aPC approach provides ability to model complex systems with unknown probability distribution functions, when only data sets of limited size or prior knowledge is available. The primary goal was to demonstrate the application and feasibility of aPC-based methods in the context of realistic CO_2_ injection scenarios. We implemented this method with the base case Kimberlina storage scenario. Five uncertain parameters with assumed uncertainty distributions are used to compute the mean of above zone pressure buildup and the associate deviations in prediction related to the model uncertainties. The results show large uncertainties in the above zone pressure prediction, making it important to analyze the role of each individual parameter on the output space. Also, it emphasizes the need for site-specific data for efficient model predictions. The above zone pressure sensitivity to different geological parameters is then evaluated and quantified using Sobol indices. The results have shown that the most influential parameter for the pressure buildup responses is the permeability of the AZMI layer. The other parameters have almost equal influence on the predictions with different trends over time. Since, in general, the involved input uncertainties are hypothetical and just for demonstration purposes, the implications of this study are limited to the probabilistic assumptions made in this study and might not truly represent the actual CO_2_ storage system.

## Additional Information

**How to cite this article**: Namhata, A. *et al*. Probabilistic Assessment of Above Zone Pressure Predictions at a Geologic Carbon Storage Site. *Sci. Rep.* **6**, 39536; doi: 10.1038/srep39536 (2016).

**Publisher's note:** Springer Nature remains neutral with regard to jurisdictional claims in published maps and institutional affiliations.

## Supplementary Material

Supplementary Information

## Figures and Tables

**Table 1:** Reference parameters used for this study: horizontal permeability (*k*_*h*_), anisotropy ratio (*k*_*v*_/*k*_*h*_), porosity (*Φ*), pore compressibility (*β*_*p*_), van Genuchten parameters (*α, m*), Brooks-Corey parameter (*γ*), bubbling pressure (*P*_*b*_), residual brine saturation (*S*_*rb*_) and residual CO_2_ saturation (*S*_*rc*_).

Parameter	Reservoir	Caprock	AZMI
*k*_*h*_(mD)	depth dependent*	0.002	0.1
*k*_*v*_/*k*_*h*_	0.2	0.5	0.5
*Φ*	depth dependent*	0.338	0.32
*β*_*p*_ (10^−10^ Pa^−1^)	4.9	14.5	14.5
*α* (10^−5^ Pa^−1^)	13	0.42	—
*m*	0.457	0.457	—
*γ*	—	—	2
*P*_*b*_	—	0.01	0.02
*S*_*rb*_	0.30	0.45	0.35
*S*_*rc*_	0.25	0.40	0.30

^*^Depth dependent values are taken from Wainwright *et al*.^30^.

**Table 2:** Second-order Sobol indices for five parameters: [1] AZMI permeability (k_AZMI_), [2] AZMI porosity (Φ_AZMI_), [3] thickness of AZMI (H_AZMI_), [4] caprock permeability (k_caprock_) and [5] caprock thickness (H_caprock_) at coordinates above the injection well, i.e. coordinate (34, 46) and, 4 km away from injection well southwards, i.e., coordinate (30, 44) at the end of injection (=50 years).

Sobol index	Value at (34, 46)	Rank at (34, 46)	Value at (30, 44)	Rank at (30, 44)
S_1_	0.215	4	0.285	2
S_2_	0.171	5	0.467	1
S_3_	0.397	1	0.165	3
S_4_	0.268	3	0.081	6
S_5_	0.303	2	0.073	7
S_1–2_	0.042	8	0.102	4
S_1–3_	0.055	7	0.037	8
S_1–4_	0.003	10	0.003	11
S_1–5_	0.001	14	0.002	13
S_2–3_	0.017	9	0.085	5
S_2–4_	0.001	13	0.004	10
S_2–5_	0.001	15	0.003	12
S_3–4_	0.002	11	0.001	14
S_3–5_	0.001	12	0.001	15
S_4–5_	0.073	6	0.006	9
