# Supplementary material for: Probabilistic Assessment of Above Zone Pressure Predictions at a Geologic Carbon Storage Site
Source: Sci Rep. 2016 Dec 20;6:39536. doi: 10.1038/srep39536 (PMC5172198; doi:10.1038/srep39536)
Supplement: Supplementary Information [file srep39536-s1.pdf]

Supplementary Information

Probabilistic Assessment of Above Zone Pressure Predictions at a Geologic Carbon Storage Site

Argha Namhata[1, 2, *], Sergey Oladyshkin[3], Robert M. Dilmore[2], Liwei Zhang[2], David V. Nakles[1]

1 Department of Civil & Environmental Engineering, Carnegie Mellon University, Pittsburgh, PA 15217, USA

2 U.S. Department of Energy, National Energy Technology Laboratory, 626 Cochrans Mill Road, Pittsburgh, Pennsylvania 15236, USA

3 Department of Stochastic Simulation and Safety Research for Hydrosystems (IWS/SRC SimTech), University of Stuttgart, Germany

** Corresponding Author: Email: anamhata@andrew.cmu.edu, Tel: +1 (412) 370-4671*

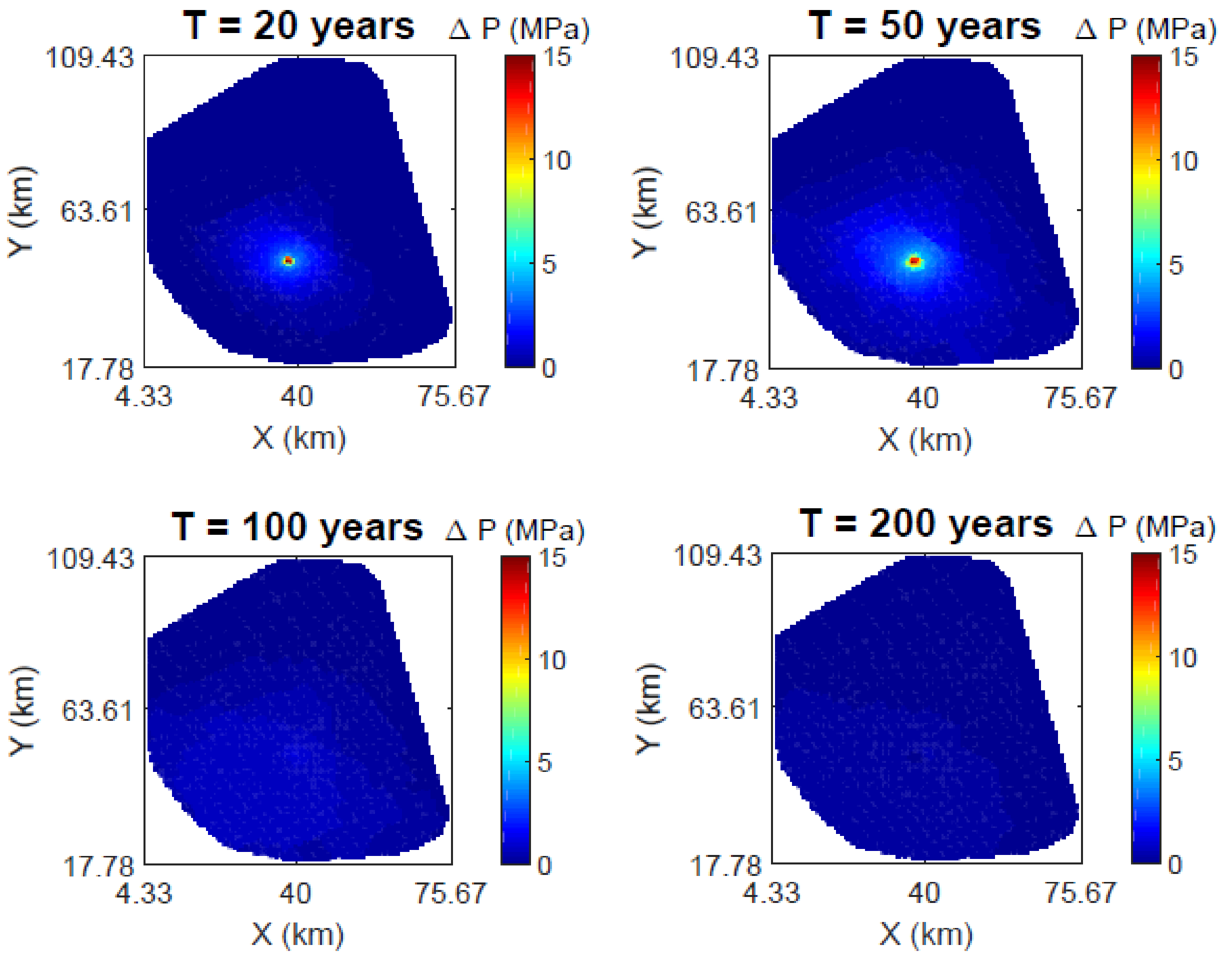

Figure S1: Evolution of pressure buildup (in MPa) at the top of the reservoir at 20, 50, 100 and 200 years after the start of CO_2 injection.

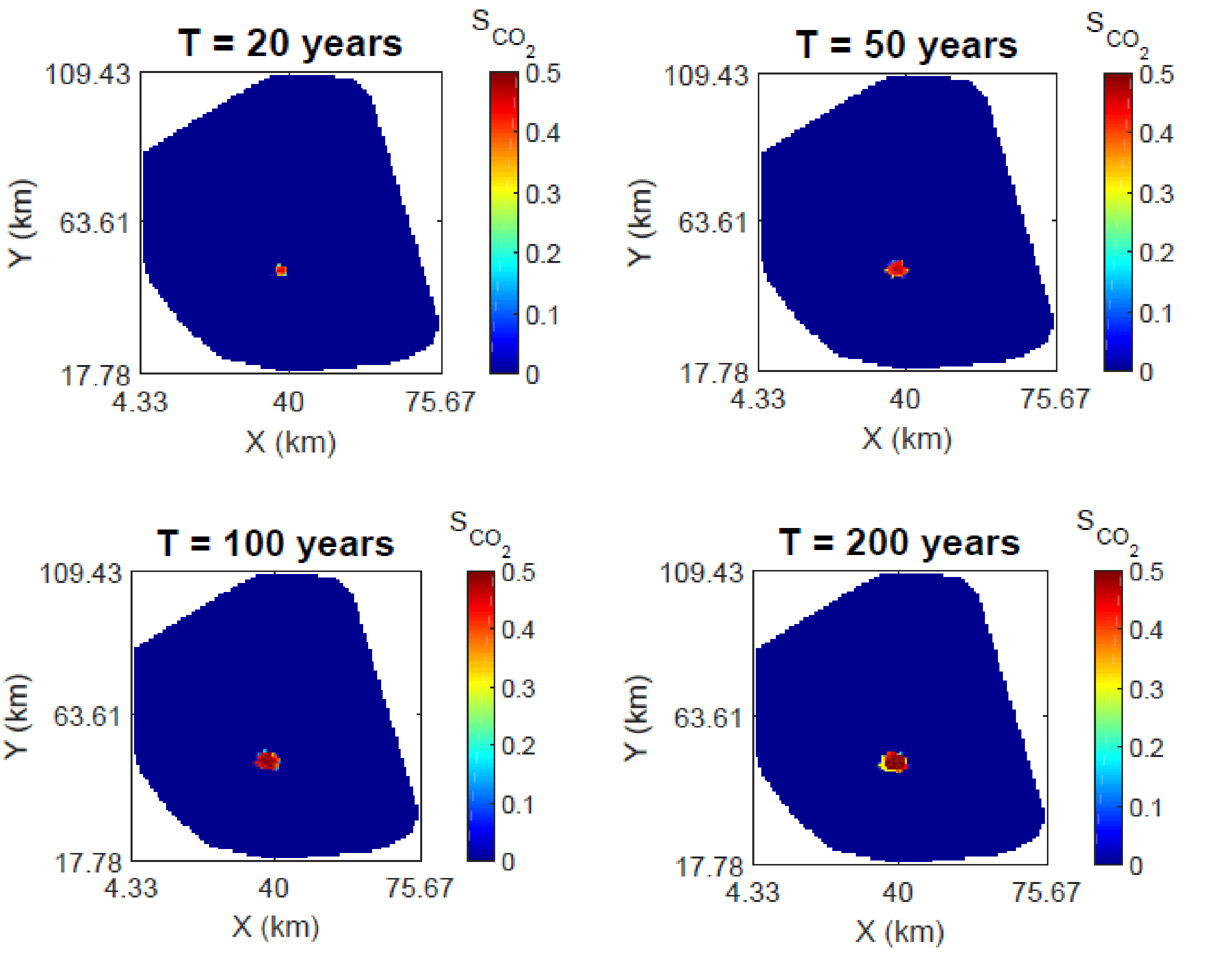

Figure S2: Evolution of CO_2 saturation at the top of the reservoir at 20, 50, 100 and 200 years after the start of CO_2 injection.

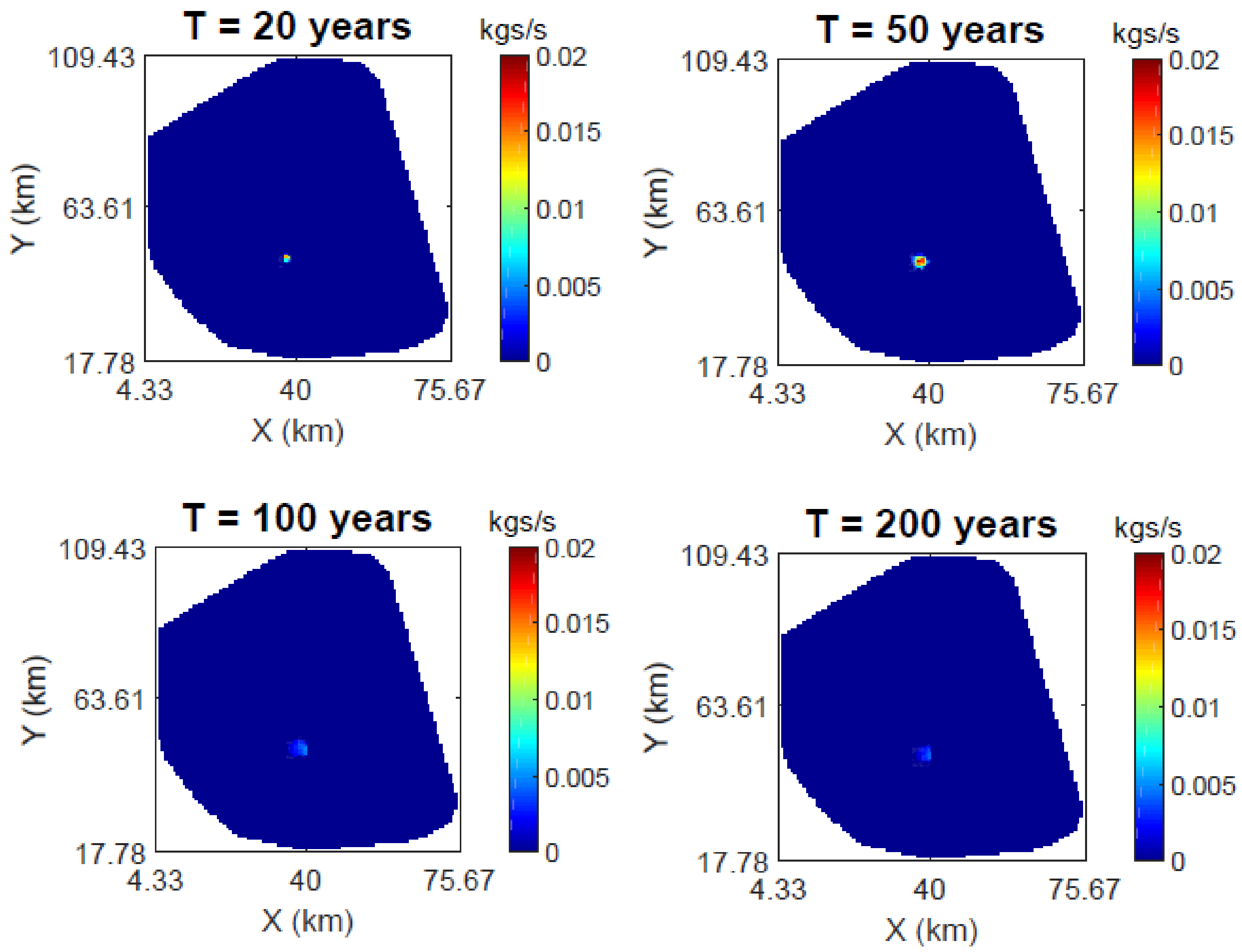

Figure S3: CO_2 flux evolution at the top of the caprock at 20, 50, 100 and 200 years after the start of CO_2 injection.

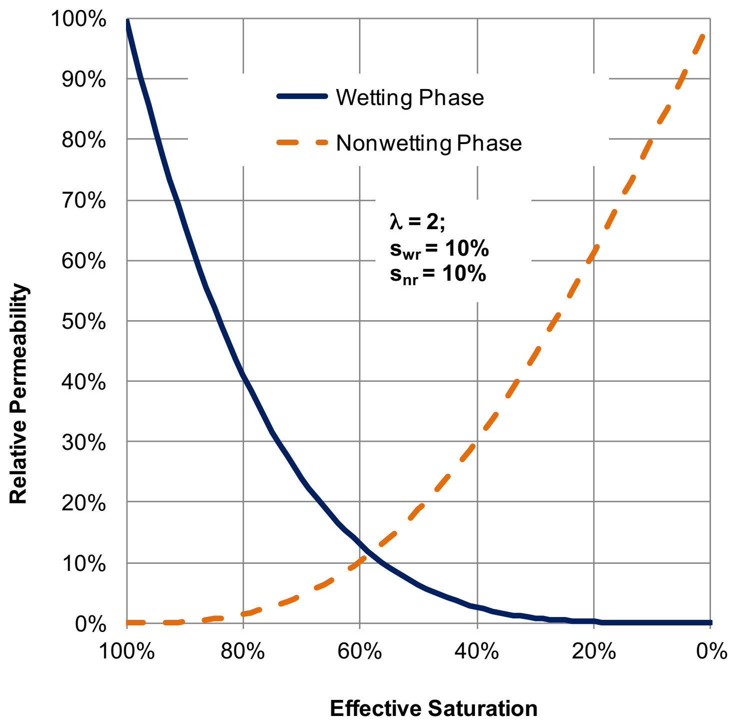

Figure S4: Brooks-Corey relationship of relative permeabilities to effective (wetting) saturation where λ is the Brooks-Corey parameter; S_{wr} is the residual wetting phase saturation and S_{nr} is the residual non-wetting phase saturation.

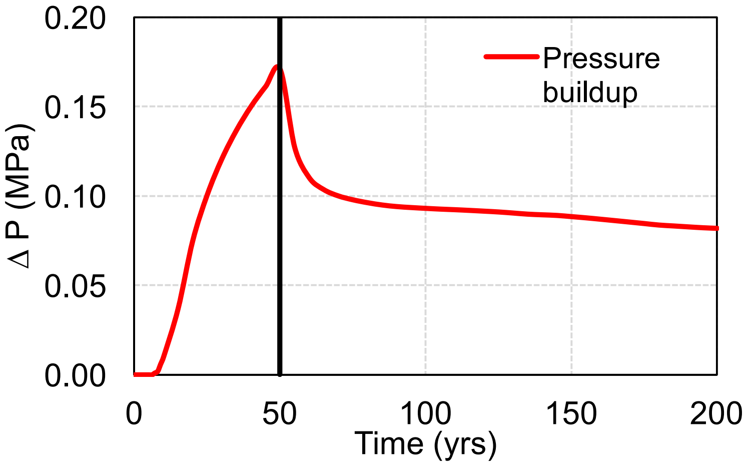

Figure S5: Time evolution of pressure buildup (in MPa) at the top of AZMI above the injection well, i.e., coordinate (34,46).

Arbitrary Polynomial Chaos Expansion

For simplicity, only one random variable ω_j is considered from the vector $\omega = \{\omega_1, ..., \omega_N\}$ in the following explanation. The basis of degree *d* for parameter ω_j is defined as $\{P_j^{(0)}, ..., P_j^{(d)}\}$. The polynomial $P_j^{(k)}(\omega_j)$ of degree *k* in the variable ω_j is defined as:

$$P_j^{(k)}(\omega_j) = \sum_{i=0}^{k} p_{i,j}^{(k)} \omega_j, \quad k = \overline{0,d}, \quad j = \overline{0,N}. \tag{S-1}$$

where,

$p_{i,j}^{(k)}$ are coefficients in $P_j^{(k)}(\omega_j)$.

The coefficients $p_{i,j}^{(k)}$ are constructed in such a way that the polynomials in equation (S-1) form an orthogonal basis in arbitrary data distributions. These arbitrary data distributions can be in any discretized, continuous, or discretized continuous form and can be quantified using any generalized statistical format.

The unknown polynomial coefficients $p_{i,j}^{(k)}$ are defined using the following matrix equation[9]:

$$\begin{bmatrix} \mu_{0,j} & \mu_{1,j} & .... & \mu_{k,j} \\ \mu_{1,j} & \mu_{2,j} & .... & \mu_{k+1,j} \\ .... & .... & .... & .... \\ \mu_{k-1,j} & \mu_{k,j} & .... & \mu_{2k-1,j} \\ 0 & 0 & .... & 1 \end{bmatrix} \begin{bmatrix} p_{0,j}^{(k)} \\ p_{1,j}^{(k)} \\ .... \\ p_{k-1,j}^{(k)} \\ p_{k,j}^{(k)} \end{bmatrix} = \begin{bmatrix} 0 \\ 0 \\ .... \\ 0 \\ 1 \end{bmatrix}. \tag{S-2}$$

Here $\mu_{i,j}$ are the non-central statistical moments of order *i* for random variable ω_j.

For further analysis we will employ the normalized orthogonal basis that has useful properties. This orthonormal basis is obtained as:

$$\hat{P}_j^{(k)}(\omega_j) = \frac{P_j^{(k)}}{||P_j^{(k)}||} \tag{S-3}$$

Where, the norm of the polynomial || $P_j^{(k)}$ || for space of events Λ (where $\omega_j \in \Lambda$) with probability measure Γ defined as:

$$||P_j{}^{(k)}||^2 = \int_{\omega_j \in \Lambda} \left[P_j{}^{(k)}(\xi)\right]^2 d\Gamma(\omega_j) \qquad \text{(S-4)}$$
